# Perception versus reality: analysis of time spent on bedside rounds in an academic ICU (Intensive Care Unit)

**DOI:** 10.1186/s12909-023-04243-y

**Published:** 2023-04-21

**Authors:** Haroon Ahmed, Austin B. King, Nada Mohamed, Erica Mokaya, Kyle Chapman, Rachel Leonard

**Affiliations:** West Virginia University Health Sciences Center, Morgantown, WV USA

**Keywords:** Graduate Medical Education, Medical Education, Bedside teaching, ICU bedside teaching

## Abstract

**Background:**

Despite its importance, teaching at the bedside is declining over time. This purported decline has not been quantified. Quantifying bedside teaching is challenging, and we found only one study quantifying bedside teaching on a hospitalist service.

**Objective:**

We conducted a study to understand the prevalence of bedside teaching in our medical intensive care unit.

**Methods:**

We conducted a single-center single-unit study in the medical intensive care unit of an academic tertiary care institution. We used a survey tool to assess perceived time spent on bedside teaching, quality of teaching, and total rounding time. In parallel, independent observers objectively measured time spent on rounds and on bedside teaching. Residents were asked to complete the survey once a week. Independent observers collected data daily and weekly averages were obtained.

**Results:**

43 responses were collected over a 4-month period. Most respondents (73%) reported a total rounding time of either 90–120 min or greater than 120 min. Median reported bedside teaching time was 16–20 min with 16 respondents (37%) reporting less than 15 min and 27 respondents (63%) reporting 16 min or more. The amount of time spent on bedside teaching was reported as adequate or more than adequate by 77% (33) of respondents with 58% (25) reporting that bedside teaching was very or extremely effective in helping them learn. Mean census reported by the independent observers was 12.75 patients per team. Bedside teaching represented an average of 12% of total rounding time, 16.85 min per day. While total rounding time increased with increasing census, there was no decline in bedside teaching time.

**Conclusion:**

It is reported that bedside teaching has decreased over time. Our study has demonstrated that bedside teaching occurs in our Medical ICU, and though it represents a minority of the time spent on rounds, residents still reported teaching in the ICU to be adequate.

**Supplementary Information:**

The online version contains supplementary material available at 10.1186/s12909-023-04243-y.

## Introduction

Since the first residency training program under the tutelage of William Osler, bedside teaching has been an essential part of medical education [1]. Recognizing that only so much can be learned from books or lectures in a formal classroom, clinical rotations are still heavily featured in undergraduate medical education. For graduate medical education, hands-on learning represents an even larger proportion of training. The ideal breakdown of medical education is a frequent topic of debate [2, 3]. Many attending physicians have an opinion, no doubt influenced by different educational experiences that were particularly instructive during their training.

What is bedside teaching? In the context of physician education, it is medical training done with direct patient care involving a trainee (student or resident physician), a more senior provider (usually the attending physician, though a senior resident or fellow can assume this role), and the patient [4]. Bedside teaching is not limited to patients confined to the bed or the inpatient setting but must involve seeing a patient directly and include the discussion that follows. The interaction can also involve more advanced modalities such as ultrasound to enhance the exam. Additional elements may include a summative review of the case leading into a differential diagnosis, proposition of further testing to narrow the differential, and assessment of recommended treatments. Often, it will use the findings from the patient’s specific case to launch into a more theoretical discussion in medicine [5]. A recent systematic review concluded that bedside rounds appear to have a positive effect on learner behavior and healthcare delivery [6]. Ideally, the patient should be engaged throughout for both a better understanding of their health and to advocate for their autonomy by shared decision-making.

What does not constitute bedside teaching? Multi-disciplinary rounds in the ICU (Intensive Care Unit) with thorough case discussion, while effective in delivering quality care, can fail to meet the definition of bedside teaching if the patient is not involved. Due to restrictions involving isolation and the desire to preserve personal protective equipment, many patients are seen by a minimal number of providers. Table rounds or card flipping in a conference room furthers this divide. Other limitations to bedside teaching include time restrictions, [7] increased time spent charting in the electronic medical record, a perception of a diminished value for physical exam, lack of comfort in exam techniques, and a concern for patient discomfort [8].

Seemingly since the days of Osler, physicians have regretted changes in education. Multiple publications have bemoaned the decline of bedside teaching [4, 9–12]. Many publications cite a 1964 study estimating that 75% of teaching was done at the bedside [13] compared to a more recent figure of 16–17% [14, 15]. At a faculty development program featured at our institution, this idea of bedside teaching as an endangered system was reiterated. However, this observation is not congruent with our practice in the critical care setting. Indeed, a survey of Pulmonary and Critical Care fellowship program directors demonstrated that 100% of responding programs used bedside teaching often or daily. 91% also used informal teaching sessions often or daily, and 75% used didactic lectures often or daily [16]. The proportion of time spent on each teaching modality was not specified. Further literature review on bedside teaching specifically in the ICU revealed few publications.

In this study, we sought to quantify the amount of time spent on bedside teaching during ICU rounds using independent observers and assess the learners’ perception of bedside teaching through survey data. Additionally, we evaluated the proportion of morning rounds spent at the bedside in direct patient contact and assessed for any changes in time spent teaching based on ICU census. Some of the results of this study have been previously reported in the form of an abstract [17].

## Materials and methods

We conducted a prospective observational study between August 2020 and November 2020 to understand the practices surrounding medical education at the bedside in a medical intensive care unit (MICU) of a tertiary care hospital. The MICU is comprised of 28 beds and staffed by two independent teams. Each MICU team includes one attending intensivist, one pulmonary and critical care medicine fellow, three to five resident physicians from internal medicine, emergency medicine, family medicine and occasionally other departments, a pharmacist, and a nutritionist. The attending intensivists work in 7-day blocks, while the residents and fellows switch every month.

The study was composed of two arms running in parallel. Medical residents and students rotating through the medical ICU were requested to fill out an online survey. At the same time, an independent observer used a stopwatch to accurately quantify the time spent on rounding and teaching at bedside.

We designed a questionnaire on Microsoft Forms and hosted it on the institutional Office365 platform. After a brief description of bedside teaching and the scope of the study, we asked participants to provide their level of training and answer questions about time spent on rounding, total time spent on teaching, time spent on teaching at bedside, perception of quality and effectiveness of bedside teaching and an open-ended question about any suggestions or comments (See addendum 1). We used 5-point Likert scales for questions about perception of quality and effectiveness, and interval scales for “time spent” questions. A 2-dimensional “quick response” (QR) bar code that linked directly to the survey was printed and visibly posted in the MICU resident workroom. Residents and medical students on their MICU rotation were asked once a week to scan the QR code using their cellphones and fill out the survey based on their experience from the previous week.

The MICU pharmacist on each team was designated as the independent observer and instructed to discreetly and accurately measure time spent on rounds with attention to time spent on specific activities that would qualify as bedside teaching: any demonstration of physical exam findings, talking about disease processes or management principles, any procedures supervised by the attending physician at bedside including point of care ultrasound, ventilator interpretation and management. In addition, the independent observer would report on the daily AM census for each team and record this data in a spreadsheet.

To limit changes in behavior related to the Hawthorne effect, attending physicians were not informed of this study being performed. Residents, fellows, students, and pharmacists were instructed to not disclose the study to the attending physicians.

All collected survey data was automatically saved and tabulated. Independent observers tabulated data into a spreadsheet daily. Data collected from both independent observers was aggregated for ease of analysis. The relationship between total rounding time, bedside teaching time and daily census was studied using linear regression. Median survey responses on total rounding and bedside teaching time were compared with mean values from independent observer data weekly.

The institutional review board (IRB) of West Virginia University granted an IRB exemption to this study. IRB # 2007064475.

## Results

43 responses were collected over a 4-month period from August 2020 to November 2020 from a pool of about 55 interns, residents, and medical students that rotated through the MICU for that duration. 38 of the 43 responses were from PGY-1 and PGY-2 residents.

**Fig. 1:**
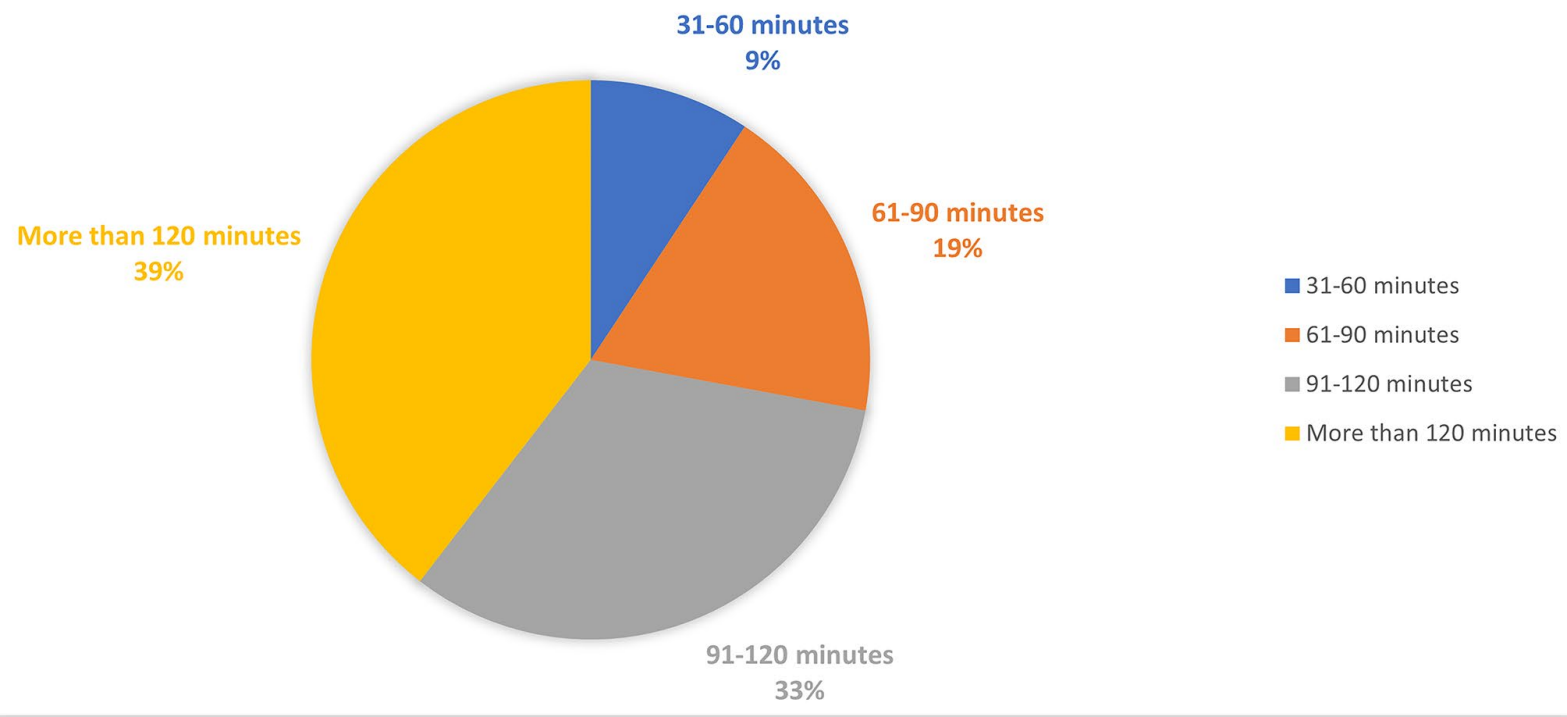
On average, how much time was spent rounding on patients with attending?

**Fig. 2:**
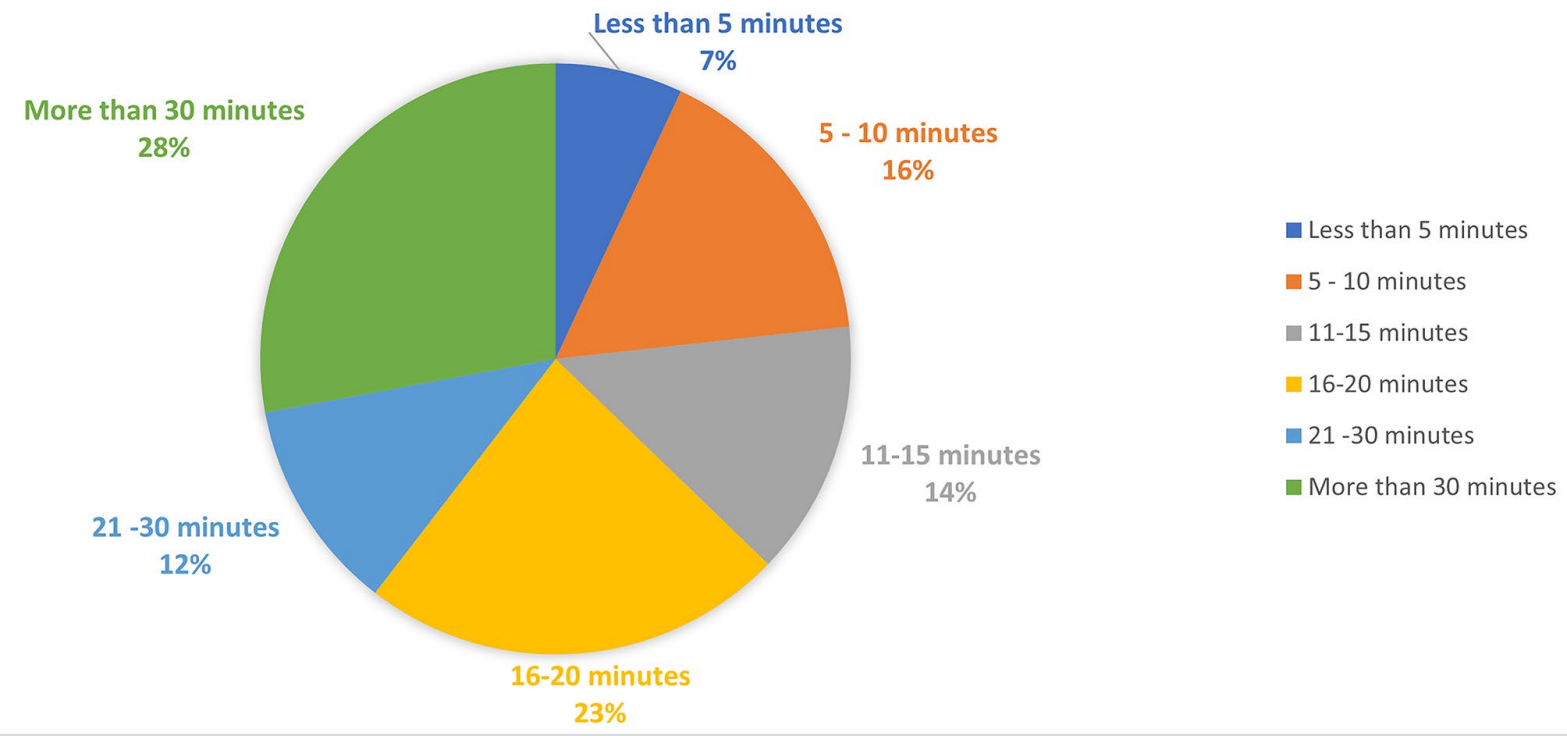
Over the past week, on average how much time was spent on bedside teaching each day?

**Fig. 3:**
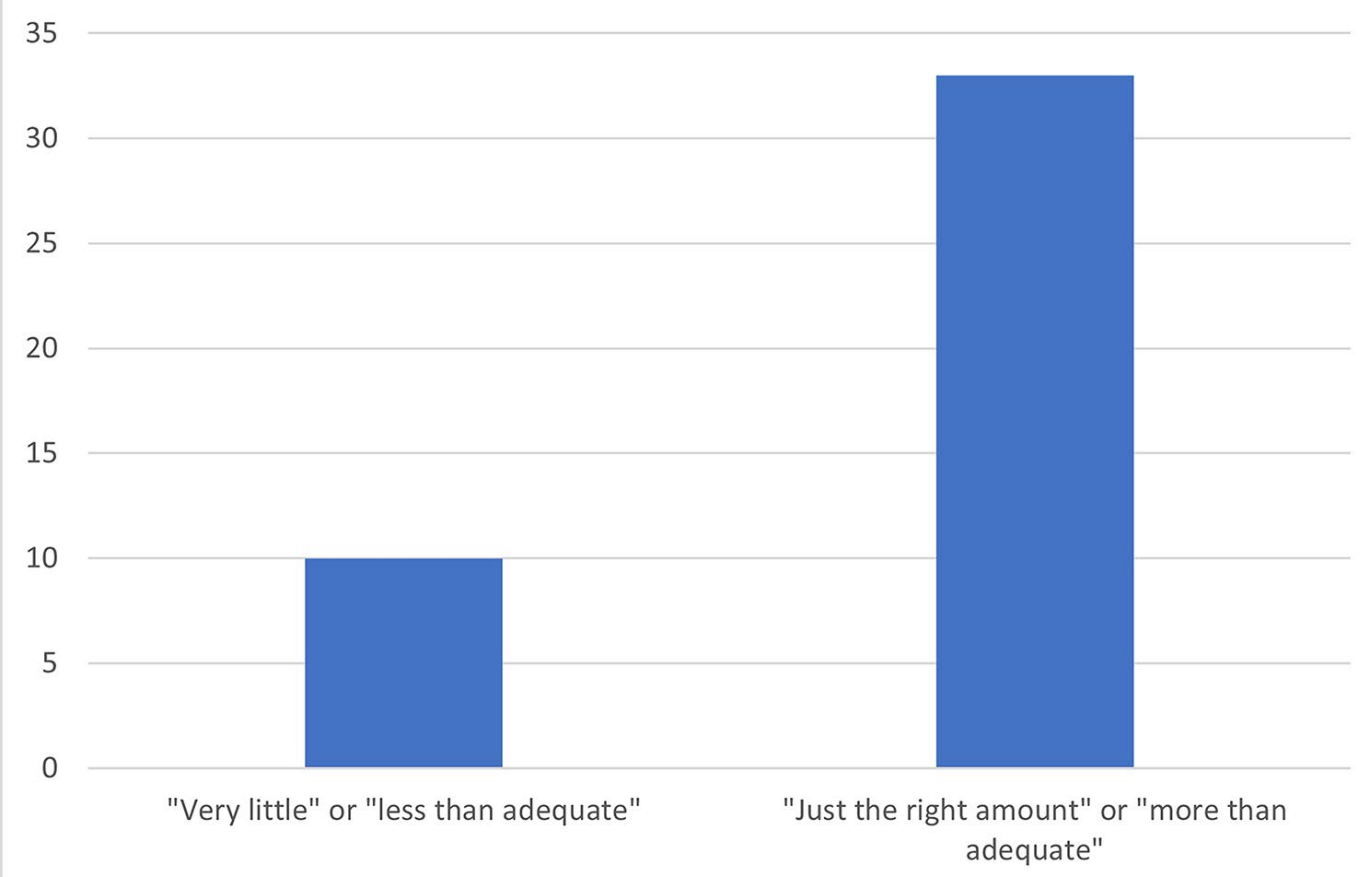
Do you think enough time was spent on bedside teaching?

**Fig. 4:**
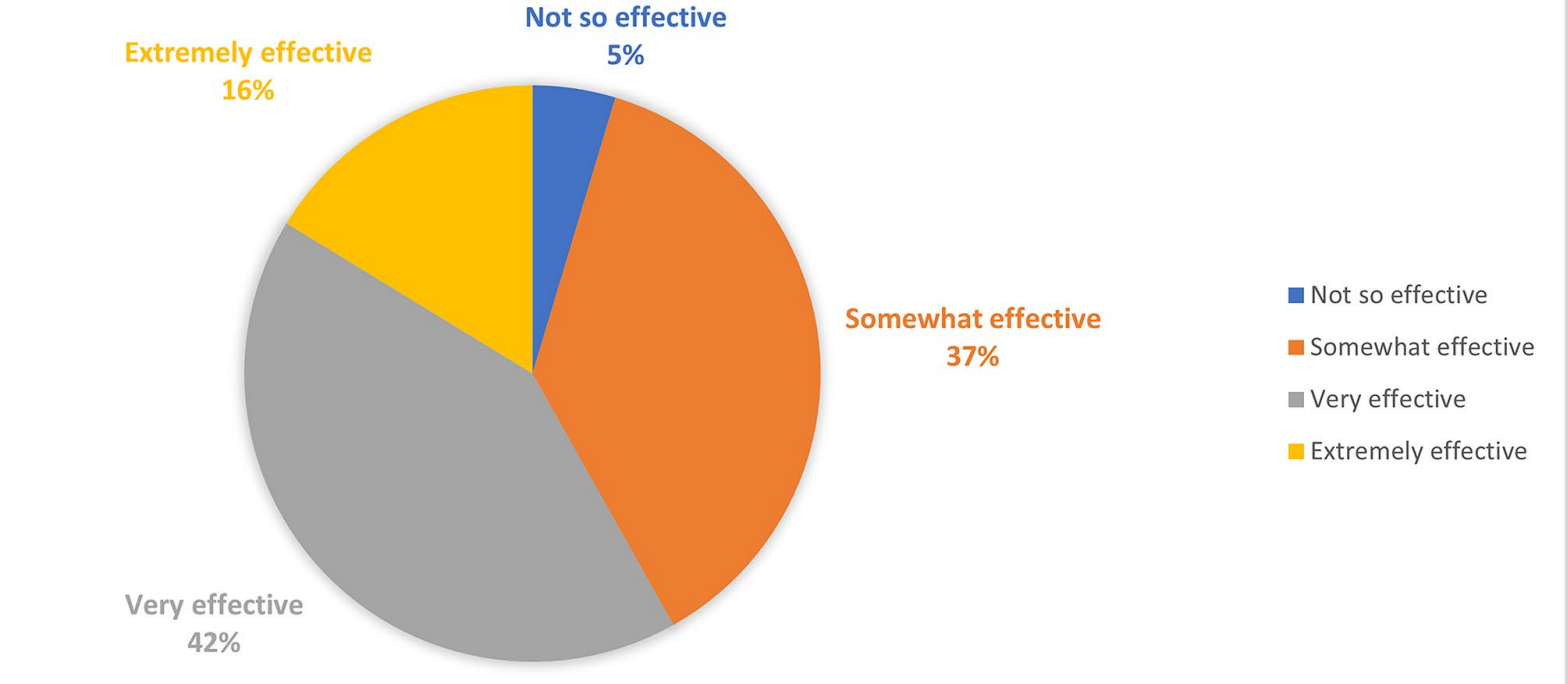
Do you think that teaching at bedside helped you learn effectively?

Most respondents (73%) reported a total rounding time of either 90–120 min or greater than 120 min (Fig. 1). Median reported bedside teaching time was 16–20 min with 16 respondents (37%) reporting less than 15 min and 27 respondents (63%) reporting 16 min or more (Fig. 2). The amount of time spent on bedside teaching was reported as adequate or more than adequate by 33 (77%) of respondents (Fig. 3) with 25 (58%) reporting that bedside teaching was very or extremely effective in helping them learn (Fig. 4). The respondents also reported they perceived most of the time spent teaching on rounds was at bedside (16–20 at bedside vs. 16–25 total teaching time).

Independent observers collected data daily except for weekends and holidays. The mean morning census reported by the independent observers was 12.75 patients per team. Bedside teaching represented an average of 12% of total rounding time at 16.85 min per day. While total rounding time increased in correlation with increased census, there was no decline in bedside teaching time with increasing census. A linear relationship was noted between survey responses and independent observers on bedside teaching time.

## Discussion

Medical education has adapted over time to gradually incorporate informal teaching sessions, simulations, and mini lectures in addition to traditional bedside teaching. Despite the evolution in medical education, bedside teaching remains an integral part of post-graduate medical education. A recent systematic review evaluated learning outcomes related to bedside teaching and most studies found that bedside teaching was useful, improved communication and encouraged ongoing attempts to facilitate its use in medical education [6]. Previous studies have suggested a decrease in bedside teaching quantity given increases in other clinical demands, lack of comfort, and fear of exposing inadequacy [18, 19].

Analysis of our results showed that the total average bedside teaching time by attending physicians on rounds was 17 min per day which represented 12% of total rounding time. This is on par with current estimates and other recent studies evaluating the quantity of bedside teaching [ 14, 15, 20]. The total time of bedside rounds was consistent despite increasing patient load and rounding time. This does indicate that the total time per patient decreased with increasing census but demonstrated persistent commitment to bedside teaching by the ICU physicians despite time limitations.

While 12% of the recorded time spent on bedside education may seem like a low proportion of morning rounds, medical residents perceived the time and quality of bedside teaching to be adequate. With advances in modern medicine, there are now many more ways to learn and teach than in the days of Osler. Particularly in the age of COVID-19, many residency programs have adapted their curriculum to embrace more virtual options [21]. To illustrate this broad range of learning, consider the experience of a resident spent in the medical ICU at our institution: daily didactic lectures from internal medicine (in- person and virtually), recurring small group simulation sessions (both for procedures and situation-oriented cases), multiple daily 10–15 min talks on patient-specific diseases during rounds, monthly review of the latest publications in the medical literature via journal club, and in the afternoon an informal session with the ICU faculty or fellow at least weekly to review ICU-specific subjects in more depth (ventilators, vasopressors, sedation and analgesia). Beyond these methods, many critical care attendings incorporate ultrasound training, ventilator wave form demonstration, blood gas analysis, and chest x-ray interpretation into daily rounds. Given such an abundance of education, there is a risk of decreasing time spent with the patients [21]. This study did not evaluate these other forms of teaching that occur during the day and did not consider other educators within the team (fellows teaching residents, residents teaching residents, etc.). Any of these factors may be why residents rated the time and quality of education highly.

The study had several strengths. The data was independently obtained by our pharmacy colleagues on rounds. This data was then cross-referenced with resident data about the amount of time that was spent teaching on rounds and impression of the quality of teaching. The attending physicians were blinded to the study occurrence, knowing neither the details of the study timeframe nor that bedside teaching was being evaluated. This study was also conducted over 4 months, capturing the bedside teaching of many different physicians and found the level of bedside teaching to be consistent throughout the department.

There are several limitations for this study. This study was limited to one medical intensive care unit in an academic tertiary care hospital. This study was conducted during the COVID-19 pandemic which may have affected results, although the number of COVID-19 patients in the state and hospital stayed low until October-November 2020. Due to changes in visitation policy, no families were present in the ICU during this time. In the ICU, bedside teaching includes modeling of family discussions and, as a result, this may have impacted the total time of bedside teaching [10]. Without the pandemic-related limitations for visitors, time spent at the bedside for family discussions would have been even higher than in our study. The impact of COVID-19 on bedside teaching has been acknowledged elsewhere [22].

This study is also observational, and survey based, which could lead to bias. However, median bedside education time on survey and independent observer data was not significantly different, arguing against bias. We also did not assess the inter-rater reliability of our pharmacists prior to conducting the study, which may have affected results. The exclusion of weekends and holidays may also have affected the true reflection of bedside teaching, but since the team structure is mostly unchanged on the weekend, it was unlikely to have fluctuated significantly. Over the weekend and on holidays, there is less time constraint due to the lack of noon didactic conferences. As a result, there may have been increased bedside teaching during these times. We also acknowledge that learner perception of quality is only the 1st Kirkpatrick level of learning evaluation [23]. Despite conducting the study over 4 months, the response rate was low, which may have led to survey bias. As discussed previously, residents considering other forms of teaching into overall perception of teaching may have elicited bias into the survey data. Use of other methods for assessing outcomes related to bedside teaching in future studies may allow for additional information regarding patient outcomes, acquisition of knowledge and/or change in behavior.

The study results are generalizable to academic intensive care units but may not be as transferable to other units due to differences in workflows and rounding styles. There is evidence that there is a decrease in bedside medical education over time. However, our study supports that bedside teaching is alive and well within the medical ICU. Given the success of bedside education, the medical ICU could serve as a model moving forward in studying bedside education and implementing other types of education. Our study also provides additional baseline information for future studies to explore optimal time spent at the bedside as well as ways to engage faculty to increase bedside teaching skills.

Further research could be done with an intervention of one of the ICU teams participating in a lecture series on the importance of bedside teaching and tips to improve this important form of education. This could be compared to a standard group to evaluate both attending and resident perception of bedside teaching as well as the total time spent teaching compared to the control group. Such studies have been done with Internal Medicine house staff and the intervention improved attending confidence as well as increased time spent in bedside teaching and residents found the intervention favorable [24, 25]. Similar studies could also be conducted in different settings to provide a greater breadth of information regarding bedside teaching over multiple different types of hospitals (community vs. academic) and ICU models (open vs. closed) as well as increase the amount of survey data available.

## Conclusion

It is reported that bedside teaching has decreased over time. However, our study has shown that bedside teaching occurs in our Medical ICU and is on par with prior estimated and measured amount of bedside teaching. Although the time teaching is a minority of the time spent on rounds, residents still reported teaching in the ICU to be adequate. Further studies could delineate the value of other teaching modalities implemented in the ICU. Additional research could involve an intervention to increase quality or quantity of bedside teaching, comparison of other units within the hospital, and evaluation of other hospitals for differences and similarities.

## Electronic supplementary material

Below is the link to the electronic supplementary material.

Supplementary Material 1

Supplementary Material 2

## Data Availability

All data generated or analyzed during this study are included in this published article [and its supplementary information files].
