# Supplementary material for: Perception versus reality: analysis of time spent on bedside rounds in an academic ICU (Intensive Care Unit)
Source: BMC Med Educ. 2023 Apr 21;23:274. doi: 10.1186/s12909-023-04243-y (PMC10120486; doi:10.1186/s12909-023-04243-y)
Supplement: Supplementary file 2 — Supplementary Material 2 [file 12909_2023_4243_MOESM2_ESM.pdf]

Introduction

Bedside teaching is any time spent teaching in the patient room, at the patient's bedside. Examples of this would be discussing physical exam findings, teaching ultrasound techniques, ventilator interpretation and management, teaching procedural skills, discussing clinical knowledge etc.
This survey is intended to understand how much time is spent at the bedside for teaching fellows, residents and medical students.

1. What is your current training level?

- ◯ MSIII-MSIV
- ◯ PGY1
- ◯ PGY2
- ◯ PGY3
- ◯ PGY4
- ◯ Other

2. On average, how much time was spent rounding on patients with attending?

- ◯ 0-30 minutes
- ◯ 31-60 minutes
- ◯ 61-90 minutes
- ◯ 91-120 minutes
- ◯ More than 120 minutes

3. Over the past week, on average how much time was spent on bedside teaching each day?

- ◯ Less than 5 minutes
- ◯ 5 - 10 minutes
- ◯ 11-15 minutes
- ◯ 16-20 minutes
- ◯ 21 -30 minutes
- ◯ More than 30 minutes

4. Do you think time spent on rounds was adequate?

- ◯ Too short and rushed
- ◯ On the short side, but adequate
- ◯ Not too long or too short
- ◯ On the longer side, but adequate
- ◯ Too long and exhausting

5. Do you think enough time was spent on bedside teaching?

- ◯ Very little
- ◯ Less than adequate
- ◯ Just the right amount
- ◯ More than adequate
- ◯ Too much

6. Do you think that teaching at bedside helped you learn effectively?

- ◯ Not at all effective
- ◯ Not so effective
- ◯ Somewhat effective
- ◯ Very effective
- ◯ Extremely effective

7. Overall, how much time did your attending spend on teaching on rounds AND at bedside?

- ◯ Less than 5 minutes
- ◯ 5-15 minutes
- ◯ 16-25 minutes
- ◯ 26-30 minutes
- ◯ More than 30 minutes

8. What suggestions do you have to improve time and quality of teaching on rounds?

This content is neither created nor endorsed by Microsoft. The data you submit will be sent to the form owner.

Microsoft Forms
